# Risk factors for preterm labor: An Umbrella Review of meta-analyses of observational studies

**DOI:** 10.21203/rs.3.rs-2639005/v1

**Published:** 2023-03-14

**Authors:** Ioannis Mitrogiannis, Evangelos Evangelou, Athina Efthymiou, Theofilos Kanavos, Effrosyni Birbas, George Makrydimas, Stefania Papatheodorou

**Affiliations:** General Hospital of Arta; University of Ioannina; Guy’s and St Thomas’ NHS Foundation Trust; University of Ioannina; University of Ioannina Medical School; University Hospital of Ioannina; Harvard Chan School of Public Health, Harvard University, Cambridge, MA, USA

## Abstract

Preterm birth defined as delivery before 37 gestational weeks, is a leading cause of neonatal and infant morbidity and mortality. Understanding its multifactorial nature may improve prediction, prevention and the clinical management. We performed an umbrella review to summarize the evidence from meta-analyses of observational studies on risks factors associated with PTB, evaluate whether there are indications of biases in this literature and identify which of the previously reported associations are supported by robust evidence.

We included 1511 primary studies providing data on 170 associations, covering a wide range of comorbid diseases, obstetric and medical history, drugs, exposure to environmental agents, infections and vaccines. Only seven risk factors provided robust evidence. The results from synthesis of observational studies suggests that sleep quality and mental health, risk factors with robust evidence should be routinely screened in clinical practice, should be tested in large randomized trial. Identification of risk factors with robust evidence will promote the development and training of prediction models that could improve public health, in a way that offers new perspectives in health professionals.

## Introduction

Preterm Birth (PTB) is defined as delivery before 37 gestational weeks and is a leading cause of infant morbidity and mortality [1-4]. 15 million babies are estimated to be born preterm every year and the PTB rate ranges between 5–18% worldwide [3] (PTB rates in USA:12–13% [1, 2]; in Europe is 5–9% [2]). Advances in neonatology and the administration of corticosteroids before birth have improved significantly the prognosis of babies born preterm. In contrast, although vigorous research, costing millions of dollars, was carried out during the last 40 years, focusing in the prediction and prevention of preterm birth its incidence remains relatively unchanged. The most probable explanation is that preterm birth is a syndrome, rather than a single disease and many different causes may be responsible [161].

Numerous systematic reviews and meta-analyses have assessed various, non-genetic risk factors of preterm labor. Several environmental and clinical parameters such as present pregnancy characteristics, previous pregnancy history [4], infections [7, 8], environmental exposures, pharmaceutical factors [9, 10] and surgical interventions have been proposed as plausible factors related to PTB. Identifying robust risk factors for PTB should either help us define a study population for specific interventions, allocate available resources effectively and allow risk-specific treatment and understanding the mechanism leading to PTB [1]. However, the exact causes of this syndrome are still mostly unknown and the contribution of every risk factor in terms of prevention is still questionable.

To our knowledge there is no previous effort to summarize existing evidence of meta-analyses of non-genetic risk factors for PTB. We conducted an umbrella review across published meta-analyses of observational studies with the goal to map the existing evidence and critically evaluate the reported associations applying stringent criteria that assess potential systematic biases and we highlight previously studied associations that provide robust evidence of association.

## Results

### Description of Eligible meta-analyses

**Figure 1:**
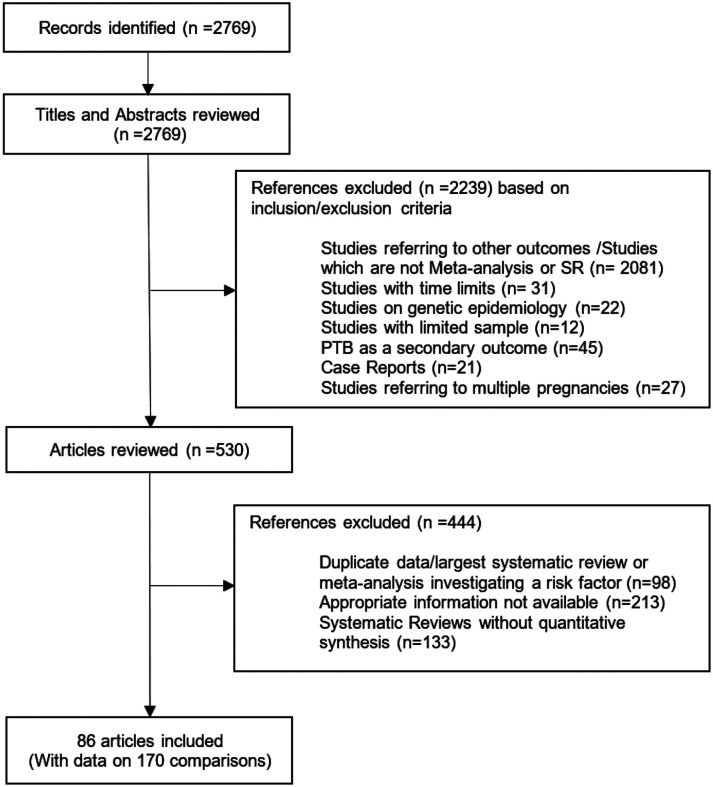
Flow Diagram for the selection of included studies

The search identified 2769 items, of which 2239 were excluded after review of the title and abstract (Fig. 1, PRISMA Flowchart). Of the remaining 530 articles that were reviewed in full text, eight articles did not report the appropriate information for the calculation of excess of statistical significance (either because the total sample size was missing or the study-specific relative risk estimates were missing), and 98 articles were excluded because a larger systematic review or meta-analysis investigating the same risk factor was available. From the 223 comparisons, we further excluded the ones that included one or two studies (53 comparisons). Therefore, 219 articles were analyzed, of which 133 were systematic reviews without any quantitative component and 86 were meta-analyses. The 86 eligible meta-analyses [26-109, 116-118] included data on 170 comparisons and 1511 primary studies.

### Summary Effect-sizes And Significant Findings

Three to 152 studies, with a median of nine studies, were included per meta-analysis. The median number of case and control subjects in each study was 88 and 529, respectively. The median number of case and control subjects in each meta-analysis was 98 and 807, respectively. The number of cases was greater than 1000 in 98 comparisons. Overall, 578 (45%) individual studies observed nominally statistically significant results. 40 meta-analyses used the Newcastle–Ottawa Scale to assess qualitatively the included primary studies. One meta-analysis used assessment criteria for non-randomized observational studies adapted from Duckitt and Harrington, 3 meta-analyses used the Methodological Index for Non-Randomized Studies (MINORS) and 38 meta-analyses used other assessment tools. Four meta-analyses did not perform any quality assessment. Details of the 170 comparisons that included 1511 individual study estimates are summarized in Supplemental Table 1.

Of the 170 comparisons, 100(58,8%) had nominally statistically significant findings at P< 0.05 using the random-effects model, of which 94 reported an increased risk and six a decreased risk for preterm birth (preconception care vs no care, magnesium supplementation vs placebo ,single vs double embryo transfer, high gestational weight gain vs normal gestational weight gain, IPI following miscarriage< 6m vs > 6m, greenery including only a 100-m NDVI buffer). Of these, a total of 62(36,5%) associations presented statistically significant effect at P < 0.001, while only 41 (24,1%) remained significant after the application of a more stringent P-value threshold of P < 10^−6^ (Supplemental Table 2)

### Between-study Heterogeneity And Prediction Intervals

Forty-five (26,5%) comparisons had large (I2 ≥ 50% and ≤ 75%) and forty-nine comparisons (28,8%) had very large (I2 > 75%) heterogeneity estimates (Supplemental Table 1). When calculating the 95% Pis, the null value was excluded in only thirty two (18,8%) comparisons.

### Small-study Effects

Evidence for statistically significant small-study effects (Egger test P < 0.10 and random-effects summary estimate larger compared with the point estimate of the largest study in the meta-analysis) was identified in 41 (24,1%) comparisons (Supplemental Table 1).

### Test Of Excess Statistical Significance

Evidence of excess-statistical-significance bias were observed in 12(7%) associations, with statistically significant (P < 0.05) excess of positive studies under any of the three assumptions for the plausible effect size, i.e. the fixed-effects summary, random-effects summary or results of the largest study (Supplemental Table 1). In addition, the observed and expected number of positive studies showed that, overall, the excess of positive results was driven by meta-analyses with large estimates of heterogeneity (I2 > 50%).

### Grading Of Evidence

The summary of the epidemiological credibility for 170 associations of risk factors for PTB is shown in Supplemental Table 1. Seven of the 170 associations (4.1%) were supported by robust evidence (fetus with isolated single umbilical artery, maternal personality disorder, sleep-breathing disorder, prior induced termination of pregnancy with vacuum aspiration, low gestational gain weight and interpregnancy interval following miscarriage less than 6 months) (Supplemental Table 4). 26 associations (15.3%) were supported by highly suggestive evidence (Intimate partner violence, cancer survivors, placenta previa, velamentous cord insertion, African/Black ethnicity, Aboriginal ethnicity, first trimester bleeding, unmarried women, obstetric cholestasis, severe maternal morbidity (hemorrhagic and hepatic disorders), Body Mass Index (BMI) > 40 kg/m^2^, cocaine exposure, endometriosis, prior surgical termination of pregnancy, maternal age > = 45 years, pregnancy with chronic kidney disease, underweight women, LEEP, LLETZ for CIN, any type of treatment for CIN with a cone depth of ≥ 10-12mm compared to untreated CIN, any type of treatment for CIN with a cone depth of ≥ 15-17mm compared to untreated CIN and PCOS). 16 associations (9,4%) were supported by suggestive evidence.

Regarding the environmental risk factors, higher residential greenness did not technically qualify to be categorized as robust evidence because the random effects p-value was 3.25 x 10^−6^ but fulfilled all other criteria.. The rest of the associations regarding different levels of exposure to air pollutants (PM_2,5,_ NO_2_) in all windows of exposure were classified as weak.

## Discussion

In this umbrella review we evaluated the current evidence, derived from meta-analyses of observational studies on the association between various risk factors and PTB. Overall, from the 170 associations that have been examined, only a minority had strongly significant results with no suggestion of bias, as can be inferred by substantial heterogeneity between studies, small study effects, and excess significance bias. Seven risk factors were supported by robust evidence, including amphetamine exposure, isolated single umbilical artery, maternal personality disorder, sleep disordered breathing measured with objective assessment, prior induced termination of pregnancy with vacuum aspiration compared to no termination, low gestational weight gain compared to normal weight gain, and interpregnancy interval following miscarriage less than 6 months. Several others had highly suggestive evidence including intimate partner violence and unmarried women, cancer survivors, Black race, placental complications, hemorrhagic and hepatic disorders, endometriosis, chronic kidney disease and treatments for CIN.

### Interpretation In The Light Of Evidence

Apart from risk factors that have been well incorporated in the clinical screening system, we identified a few that are not receiving the attention they should during pregnancy follow up despite the fact that they demonstrate robust evidence. The World Health Organization (WHO) encourages women who experienced a previous miscarriage to wait for a minimum of 6 months before the next conception to achieve optimal outcome and reduce obstetric complications such as preterm birth [119]. Contrary to the findings of the research on which WHO based its recommendations, some studies reported that the risk of adverse obstetric outcomes including preterm birth is lower in women who conceived less than 6 months after a pregnancy loss [120, 125, 126], while synthesizing all available data provided the same conclusion [105]. This meta-analysis included eight studies, performed two analyses: one including the study of Conde Agudelo 2004 [121] and one excluding it, and robust results were obtained after excluding the study. While this was a large retrospective study on which the WHO guidelines for delaying pregnancy for at least 6 months [119] are based, it did not differentiate between induced and spontaneous abortions and used data from many countries where induced abortion is illegal[121], therefore should be interpreted with caution. After a miscarriage, there is a very small burden on the folate reserve and thus miscarriage is not very likely to lead to folate deficiency in the postpartum period, so miscarriage and delivery later in pregnancy can have differential effects on subsequent pregnancy. This could explain the reduced risk of adverse outcomes in a short IPI after a miscarriage [122] but not after delivery. In support of this hypothesis, there is evidence to suggest that late miscarriages (after 12 weeks of gestation) are associated with worse outcomes in the subsequent pregnancy [123]. In addition, most women who attempt another pregnancy soon after a miscarriage are likely to be motivated to take better care of their health and consequently result in better pregnancy outcomes [124]. Another plausible reason may be that those who conceive soon after a miscarriage are naturally more fertile and younger and consequently have better pregnancy outcomes.

Another association with robust evidence was pregnant women with sleep breathing disorders. This meta-analysis clearly demonstrated the increased risk profile of women who experience SBD not only for preterm birth but for other pregnancy outcomes. Regarding plausible mechanisms, the association between SDB and intermittent maternal hypoxia as well as the link with conditions synonymous with impaired placental function such as pre-eclampsia suggest a multifactorial cause, with both physiologic changes associated with pregnancy and placental dysfunction involved. This robust association has clear implications for obstetric practice. First, given the rapidly increasing worldwide obesity rates, SDB is likely to become more prevalent in the pregnant population and is worthy of being screened for. Second, the increased risk for both adverse intrapartum and perinatal outcomes demonstrated in this review strongly support the need for increased surveillance of this cohort. Third, public health education programs must take into account the specific maternal and perinatal risks and promote education about the significance of obstructive sleep apnea symptoms and the need for women to discuss this with their obstetric caregivers. In alignment with this suggestion, women with personality disorders could be identified early through mental health screening, where targeted health interventions and multidisciplinary management can be implemented in order to reduce poor outcomes for the baby/child and woman. This early identification and support also have the potential to enable the prevention of maladaptive development trajectories within the mother infant relationship [128, 129]. Regarding induced termination of pregnancy with vacuum aspiration, our results should be interpreted with caution because it is unclear whether two of the five included studies come from the same population, therefore the variance of the pooled estimate may be artificially narrower.

Furthermore, it is important that clinical examination and medical history includes risk factors which are not well known, identified in meta-analysis with highly suggestive evidence. To be more specific regarding highly suggestive evidence, there were a few that are well known and used to classify pregnancies as high risk for PTB such as therapies for cervical intraepithelial neoplasia, advanced maternal age, placental pathology, race, first trimester bleeding and maternal comorbidities. There were also included factors that are not routinely screened in the obstetric population such as intimate partner violence, cancer survivors and being unmarried. When it comes to intimate partner violence exposure during pregnancy, this meta-analysis included 30 studies examining the risk of PTB [28]. Two possible pathways have been described which could lead to adverse perinatal outcomes [140, 141]. One is the direct exposure to violence consisted of either physical assault directly to the abdomen or sexual abuse. Direct exposure has been associated with pregnancy complications such as premature rupture of membranes, uterine contractions and placental damage, too[140, 142]. On the other hand, indirect exposure to violence trigger biological mechanisms, such as smoking, alcohol or drug use, inadequate prenatal care and weight gain that contribute to adverse birth outcomes [140, 145-156]. Women with history of abuse by their partner is believed to have less support, lower levels of self-esteem and higher levels of stress, too [140, 142, 145, 153, 160]. All these factors contribute to the indirect mechanism “theory” associated to preterm birth. As a result, healthcare professionals/institutes follow screening protocols in some nations or clinical guidelines, in order to detect and take care of these cases [157-159]. Another association that demonstrates highly suggestive evidence is pregnant women, whom survived cancer. This meta-analysis included fourteen studies which described the incidence of PTB [30]. Regarding plausible mechanisms, it is believed that radiotherapy treatment protocols for cancer, especially irradiation of the abdomen is harmful both for the uterine vasculature and the uterus muscular development. This leads to a reduction in uterine elasticity and uterine volume [143, 144]. Uterine volume can also be smaller due to hormonal deficiency, caused by ovarian failure [144]. This could lead to preterm delivery. However, there is a possible association between the dosage of radiotherapy and risk of PTB, something which still has not been examined due to the obscuring of pooling dosages in previous studies. Higher radiations doses may reflect to higher risk of PTB. In addition, we should highlight the fact that the population of cancer survivors following advancing treatment grows and the prevalence of PTB cases in these groups is going to rise, in regards. Maternal marital status plays also a role in PTB, but healthcare professionals rarely consider it as a risk factor. This meta-analysis consists of 21 studies comparing unmarried women to married ones, identifying an increased risk of PTB [43]. Regarding the ways in which unmarried women are associated to PTB, it is suggested that the quality of relationship between biological maternal and paternal figures is more important than their legal status [136, 137]. Moreover, a biological father might be more caring or supportive of the birth compared to another family member or partner. Mother psychosocial stress level depends on the support that she receives from her familiar environment [138, 139], but a variety of other factors should be taken into consideration before interpreting these results. With regards to health practitioner’s point of view, the importance of obtaining social history information during clinical exam, lies in identifying pregnancies at risk for PTB and offering new perspectives. This information should be focused on rarely screened factors in every-day routine, which support highly suggestive evidence.

Regarding environmental risk factors, increased residential greenness was associated with a protective effect on the risk of PTB. Although this finding was categorized as having suggestive evidence, the p-value of the random effect estimate was very close to the stringent threshold of < 10 – 6. Acknowledging the detrimental projected effect of climate change in greenness and given that it is one of the few protective risk factors for PTB, serious efforts should be made to maintain and grow residential greenness. Possible mechanisms include among others amelioration of the effects of air pollutants, reduction of stress and increase in physical activity [116]. There were also suggestive evidence for early pregnancy exposure to PM_2.5_ and the risk of PTB. This association has been debated in the literature with conflicting results about the timing and magnitude of effect and is less robust than other associations that have been shown to have strong evidence for associations [130] such as birthweight.

In the current umbrella review, we applied a transparent and replicable set of criteria and statistical tests to evaluate and categorize the level of existing observational evidence. Although, 58,8% of associations in the included meta-analyses report a nominally (P < 0.05) statistically significant random-effects summary estimate, when stringent P value was considered (P < 10^−6^), the proportion of significant associations decreased to 24,1%. 94 (55,3%) associations had large or very large heterogeneity, while when we calculated the 95% prediction intervals, which further account for heterogeneity, we found that the null value was excluded in less than half of the associations. Only seven (4.1%) of the assessed risk factors found to provide robust evidence, indicating that several published meta-analyses of observational studies in the field could be susceptible to biases and the reported associations in the existing studies are often exaggerated.

The ability to modify those factors, mainly those related to mental health and sleep quality screening, through screening and clinical interventions or public health policy measures remains to be established. Furthermore, there is no guarantee that even a convincing observational association for a modifiable risk factor would necessarily translate into large preventive benefits for preterm birth if these risk factors were to be modified [93]. With obesity becoming a global epidemic, the assessment of the strength of the evidence supporting the impact of overweight and obesity in sleep breathing disorders could allow the identification of women at high risk for adverse outcomes and allow better prevention. Obesity is generating an unfavorable metabolic environment from early gestation; therefore, initiation of interventions for weight loss during pregnancy might be belated to prevent or reverse adverse effects, which highlights the need of weight management strategies before conception [68, 103, 104, 131]. PTB does not only increase the risk for maternal and infant complications, but also significantly increases a woman’s risk of cardiovascular disease (CVD) after pregnancy, therefore primary prevention [12, 132-134] is extremely important.

Our assessment has certain limitations. Umbrella reviews focus on existing systematic reviews and meta-analyses and therefore some studies may have not been included either because the original systematic reviews did not identify them, or they were too recent to be included. In the current assessment we used all available data from observational studies, therefore the meta-analysis estimates may partly reflect the biases from which the original studies suffer from. Statistical tests of bias in the body of evidence (small study effect and excess significance tests) offer hints of bias, not definitive proof thereof, while the Egger test is difficult to interpret when the between-study heterogeneity is large. These tests have low power if the meta-analyses include less than 10 studies and they may not identify the exact source of bias [23, 25, 135]. More specifically, in our study, all robust evidence applied to meta-analyses with less than 10 studies, therefore the results of publication bias should be interpreted with caution. Furthermore, we did not appraise the quality of the individual studies on our own, since this should be included in the original meta-analysis and it was beyond the scope of the current umbrella review. However, we recorded whether and how they performed a quality assessment of the synthesized studies. Lastly, we cannot exclude the possibility of selective reporting for some associations in several studies. For example, perhaps some risk factors were more likely to be reported, if they had statistically significant results.

## Conclusion

The present umbrella review of meta-analyses identified 170 unique risk factors for preterm birth. Our analysis identified seven risk factors with robust evidence and strong epidemiological credibility pertaining to isolated single umbilical artery, amphetamine exposure, maternal personality disorder, sleep breathing disorders, induced termination of pregnancy with vacuum aspiration, low gestational weight gain and interpregnancy interval following miscarriage of less than 6 months. As previously suggested, the use of standardized definitions and protocols for exposures, outcomes, and statistical analyses may diminish the threat of biases, allow for the computation of more precise estimates and will promote the development and training of prediction models that could promote public health.

## Methods

We conducted an umbrella review which is a comprehensive and systematic approach that collects and critically evaluates all systematic reviews and meta-analyses performed on a specific research topic [11]. We used previously described, standardized methods that have been already used in previously published umbrella reviews referring to risk factors related to various outcomes [13-16] and have been elaborated below.

A protocol for this umbrella review was registered in the International prospective register of systematic review (PROSPERO 2021 CRD42021227296)

### Search Strategy

Two researchers (A.E., I.M.) independently searched PubMed database from inception to December 2020, in order to identify systematic reviews and meta-analyses of studies that examine the association between risk factors and preterm birth. The search strategy included combinations of the Medical Subject Headings (MESH) terms, key words and word variants for terms “preterm birth” AND (“systematic review” OR “meta-analysis”). Titles and abstracts were screened and potentially eligible articles were retrieved for full text evaluation. A detailed description of our search strategy is provided in the supplement (Supplemental Table 3).

### Eligibility Criteria And Data Extraction

We included systematic reviews with meta-analyses investigating the association between various types of exposures and PTB. Specifically, we included studies with singleton pregnancies and studies where PTB was evaluated as primary outcome.

Case report or series and individual participant data meta-analyses were excluded. We also excluded studies that set time limits on time span or were performed on a restricted setting (i.e. conducted for one specific country). Furthermore, we excluded studies that assessed PTB as a secondary outcome, studies including multiple pregnancies, and studies that assessed genetic or over -omics features as risk factor for PTB. All studies were compared to avoid the possibility of duplicate or overlapping samples. If more than one meta-analysis referring to the same research question were eligible, the one with the largest amount of component studies with data on individual studies’ effect sizes retained for the main analysis

Publications whom the estimates of the studied associations, such as relative risks (RR) and 95% confidence intervals (CIs), were not reported or could not be retrieved/calculated were excluded from the analysis. For the non-environmental risk factors, we also excluded meta-analyses that did not provide the number of cases in the exposed and non-exposed groups, which is used for the calculation of the excess significance tests. For the environmental risk factors, since most commonly they report the results as per unit(s) increase in exposure and everyone is exposed, we included them even if they did not report the number of cases and total sample size.

Eligible articles were screened by four independent reviewers (AE/IM and EB/TK). Any disagreement between reviewers was resolved by consensus or after evaluation of a third author (SP or EE). The data of eligible studies were extracted in a predefined data extraction form recording for each study the first author, journal, year of publication, the examined risk factors and the number of reviewed studies. Either the study specific relative risk estimates (risk ratio, odds ratio, hazard ratio, incidence rate ratio) and the confidence intervals were extracted or the mean and the standard deviation for continuous outcomes were also noted in this form. We also extracted exposed and control group used; outcome assessed; study population; exposure characteristics; number of studies in the meta-analysis; meta-analysis metric and method; effect estimate with the corresponding 95% confidence interval; number of cases and total sample size; I2 metric and the corresponding χ2 p-value for the Q test; and Egger’s regression P-value.

### Assessment Of Summary Effect And Heterogeneity

We re-calculated summary effects and 95% Confidence Intervals (CIs) for each meta-analysis via fixed and random effects model [17, 18]. 95% prediction intervals (PI) were also computed for the summary random-effects estimates, which further account for between-study heterogeneity indicating the uncertainty for the effect that would be expected in a new study examining the same correlation [19, 20]. A PI describes the variability of the individual study estimates around the summary effect size and represents the range in which the effect estimate of a new study is expected to lie.

The largest study considered as the most precise with a difference between the point estimate and the upper or lower 95% confidence interval less than 0.20. If the largest study presented a statistically significant effect, then we recorded this as a part of the grading criteria.

Between study heterogeneity was assessed and P-value of the χ^2^-based Cochran Q test and the I^2^ metric for inconsistency (reflecting either diversity or bias) was reported, too. I^2^ metric were used to indicate the ratio of between study-variance over the sum of within and between-study variances, ranging from 0–100% [21]. Values exceeding 50% or 75% are usually considered to represent large or very large heterogeneity, respectively. 95% Confidence intervals were calculated as per Ioannidis et al. [22].

### Assessment Of Small-study Effect

Small studies tend to give substantially larger estimates of effect size when compared to larger studies. We evaluated the evidence of the presence of the small study effect, in order to identify publication and other selective reporting biases. They can also reflect genuine heterogeneity, chance, or other reasons for differences between small and large studies [23]. We evaluated whether smaller (less precise) studies lead to inflated effect estimates comparted to than larger studies. We used the regression asymmetry test proposed by Egger, that examines the potential existence of small study effects via funnel plot asymmetry [24]. Egger’s test fits a linear regression of the study estimates on their standard errors weighted by their inverse variance. Indication of small study effects based on the Egger’s asymmetry test was claimed when P-value ≤ 0.10. This is considered as an indication of publication bias, Indication of small study effects based on the Egger’s asymmetry test was claimed when P-value ≤ 0.10 and the random effects summary estimate was larger compared to the point estimate of the largest (most precise) study in the meta-analysis.

### Excess Statistical Significance Evaluation

The excess significant test was applied to evaluate the existence of relative excess of significant findings in the published literature for any reason (e.g. publication bias, selective reporting of outcomes or analyses). The number of expected positive studies is estimated by a chi-squared-based test and being compared to the observed number of studies with statistically significant results (P < 0.05) [25]. A binomial test evaluated whether the number of positive studies in a meta-analysis was too large according to the power that these studies have to detect plausible effects at α = 0.05. In brief, observed versus expected studies for each meta-analysis were compared separately and this comparison also extended to groups of many meta-analysis after summing the observed and expected studies from each meta-analysis. The power of each component study was calculated using the fixed-effects summary, the random effects summary, or the effect size of the largest study (smallest SE) as the plausible effect size [15]. An algorithm using non-central t distribution was used to calculate the power of each study [26]. Excess statistical significance for single meta-analyses was claimed at P < 0.10 (one-sided P < 0.05, with observed > expected as previously proposed), given the power to detect a specific excess will be low, especially with few positive studies.[25]

### Grading Of Evidence

We followed a 4-level grading (robust, highly suggestive, suggestive and weak) to evaluate the strength of the evidence based on the following criteria: number of cases, summary random-effects P-value, between-studies heterogeneity, 95% PI, small study effects bias and excess statistical significance[110]. This grading approach based on these parameters was used because it allows for an objective, standardized classification of the level of evidence and has been previously shown that provides consistent results with other more subjective grading schemes [111, 112]. As most of the environmental risk factors included meta-analyses did not report the number of cases or the sample size of the studies included, we were unable to estimate the power of each meta-analysis and the excess significance test for these factors so we did not include excess statistical significance in the grading of these evidence.

Briefly, meta-analyses were considered to be supported by robust evidence if: the association was supported by more than 1000 cases, a highly significant association (the random effects model had a P-value ≤ 10^−6^, a threshold that is considered to substantially reduce false positive findings) [113, 114, 115], there was absence of high heterogeneity based on I2 < 50%, the 95% PI excluded the null value, and there was no evidence of small study effects or excess statistical significance. Highly suggestive evidence required more than 1000 cases, a highly significant association (a random-effects P-value ≤ 10^−6^), and the largest study in the meta-analysis was nominally significant. Associations based on metaanalyses a random-effects P-value ≤ 10^−3^ and included more than 1000 cases [113, 114, 115] were graded as suggestive evidence. The remaining nominally significant associations were graded as weak evidence (P < 0.05). We need to highlight that this specific grading scheme focuses on the reduction of false positive findings and the evaluation of potential biases in the studied associations. Therefore, the set of criteria used here is not ideal for a detailed evaluation of non-significant associations and to distinguish insufficient evidence from robust evidence of no association. That would require a different approach and another set of criteria altogether that would focus on the power of the meta-analyses to observe a significant effect, which was beyond the scope of our review.

Statistical analyses were performed using STATA version 14 (StataCorp, Texas, USA)

## Data Availability

Relevant data to our study are mainly included in the article, tables and supplemental material. However, we will share the original dataset after reasonable requests.

## Code availability:

The statistical code supporting the findings of our study will be available upon reasonable requests
